# Emerging role of non-coding RNAs in the regulation of KRAS

**DOI:** 10.1186/s12935-022-02486-1

**Published:** 2022-02-09

**Authors:** Soudeh Ghafouri-Fard, Zeinab Shirvani-Farsani, Bashdar Mahmud Hussen, Mohammad Taheri, Reza Jalili Khoshnoud

**Affiliations:** 1 Department of Medical Genetics, School of Medicine, Shahid Beheshti University of Medical Sciences, Tehran, Iran; 2 Department of Cellular and Molecular Biology, Faculty of Life Sciences and Technology, Shahid Beheshti University, Tehran, Iran; 3 Department of Pharmacognosy, College of Pharmacy, Hawler Medical University, Erbil, Iraq; 4 Institute of Human Genetics, Jena University Hospital, Jena, Germany; 5 Urology and Nephrology Research Center, Shahid Beheshti University of Medical Sciences, Tehran, Iran; 6 Skull Base Research Center, Loghman Hakim Hospital, Shahid Beheshti University of Medical Sciences, Tehran, Iran

**Keywords:** KRAS, Oncogene, lncRNA, miRNA, circRNA

## Abstract

The Kirsten ras oncogene KRAS is a member of the small GTPase superfamily participating in the RAS/MAPK pathway. A single amino acid substitution in *KRAS* gene has been shown to activate the encoded protein resulting in cell transformation. This oncogene is involved in the malignant transformation in several tissues. Notably, numerous non-coding RNAs have been found to interact with KRAS protein. Such interaction results in a wide array of human disorders, particularly cancers. Orilnc1, KIMAT1, SLCO4A1-AS1, LINC01420, KRAS1P, YWHAE, PART1, MALAT1, PCAT-1, lncRNA-NUTF2P3-001 and TP53TG1 are long non-coding RNAs (lncRNAs) whose interactions with KRAS have been verified in the context of cancer. miR-143, miR-96, miR-134 and miR-126 have also been shown to interact with KRAS in different tissues. Finally, circITGA7, circ_GLG1, circFNTA and circ-MEMO1 are examples of circular RNAs (circRNAs) that interact with KRAS. In this review, we describe the interaction between KRAS and lncRNAs, miRNAs and circRNAs, particularly in the context of cancer.

## Introduction

The Kirsten ras oncogene KRAS is a homolog from the mammalian ras gene family [1]. The encoded protein by this gene has 88 amino acid residues [2] and is a member of the small GTPase superfamily participating in the RAS/MAPK pathway. In fact, KRAS protein serves as a switching device being turned on and off by the GTP and GDP molecules. Attachment of a GTP molecule to KRAS turns this switch on leading to signal transduction. When KRAS transforms the GTP to GDP, it will become inactivated. GDP binding with KRAS stops transmission of signals to the cell nucleus. RAS/MAPK signaling pathway instructs the cell to go through proliferation stages or to differentiate into mature cells with specialized function [2]. In addition, KRAS has inherent GTPase activity which is induced by GTPase-activating proteins, mediating the direct interaction of KRAS with the effector proteins [3]. Single amino acid substitutions in *KRAS* gene has been shown to activate the encoded protein [4], resulting in cell transformation as well as resistance to a wide array of chemotherapeutics and targeted therapies against epidermal growth factor receptors (EGFRs) [5].

Mutations in RAS have been detected in approximately 15% of acute myeloid leukemia (AML), more than 10% of adult T cell acute lymphoblastic leukemia and about one third of multiple myeloma cases [6]. In some AML cases, KRAS mutations are assumed to be commencing events in the course of disease. Moreover, these mutations can occur during progression of AML [6, 7]. The presence of KRAS mutations can negatively influence overall survival and complete remission rate of these patients. In fact, KRAS mutations predict poor prognosis of AML [8]. In breast cancer, KRAS is the most commonly mutated RAS protein. Mutations in KRAs are predictor of poor prognosis and higher rate of metastatic events [9]. In colorectal cancer, RAS mutations have been detected in 45% of patients, with KRAS being the most commonly mutated one [10]. The vast majority of KRAS mutations occur at codon 12 while codon 61 harbors very few mutations [11]. A comprehensive assessment of RAS mutations in different types of cancers, including those originated from adrenal gland, autonomic ganglia, biliary tract, bone, breast, central nervous system, cervix, endometrium and hematopoietic/lymphoid system has shown that the majority of cancer types favor mutation of a single isoform, this is usually KRAS [11].

**Fig. 1:**
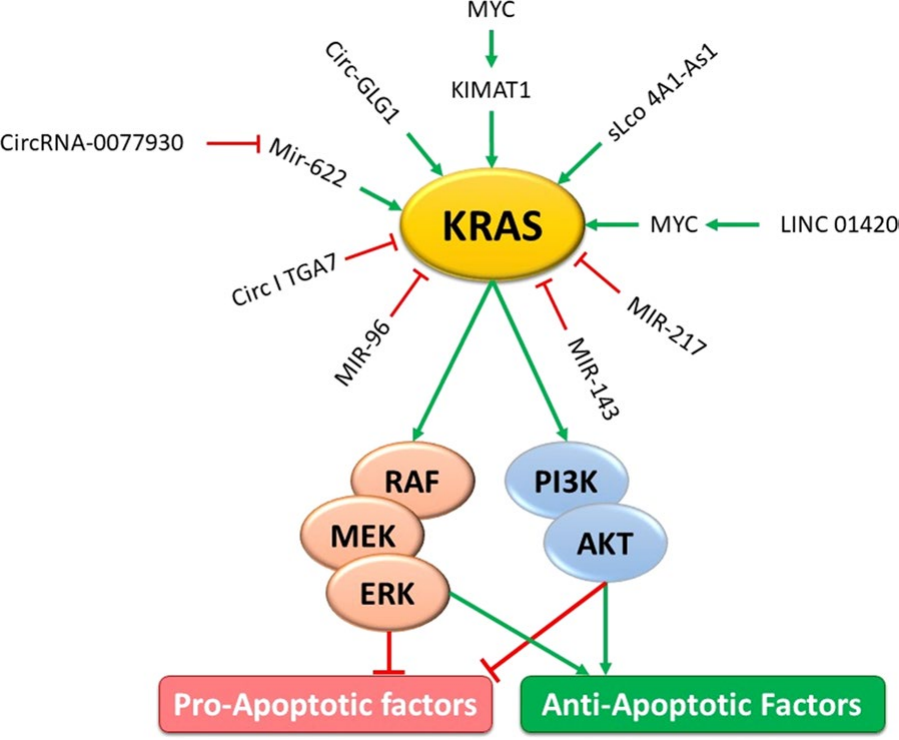
A schematic representation of the interaction between non-coding RNAs and KRAS. The expression of different noncoding RNAs including lncRNAs, miRNAs, and circular RNAs could have an important effect on KRAS expression. Various noncoding RNAs via targeting KRAS could regulate the expression of anti- and pro-apoptotic genes, thus inhibiting or promoting cell apoptosis

It has been recently evident that KRAS influence expression of a number of non-coding RNAs. Moreover, some non-coding RNAs have been found to participate in the pathogenesis of cancer through interacting with KRAS. In this review, we describe the interaction between KRAS and long non-coding RNAs (lncRNAs), microRNAs (miRNAs) and circular RNAs (circRNAs), particularly in the context of cancer. We summarized some of ncRNAs interacted with KRAS in Fig. 1.

### Interaction between lncRNAs and KRAS

LncRNA are a group of non-coding RNAs with sizes more than 200 nucleotides. These transcripts can affect expression of genes at different levels. They have diverse types of interactions with mRNAs, DNA molecules, proteins, and miRNAs and accordingly control epigenetic events and transcription of genes. Moreover, they can affect gene expression at post-transcriptional level as well as translational and post-translational phases [12]. LncRNAs interact with DNA via triple-helix formation [13].

Zhang et al. have designed an lncRNA microarray to find RAS-interacting lncRNAs. They have identified the lncRNA Orilnc1 as a downstream target of RAS that mediates oncogenic effects of RAS in cancer cell lines. They have also shown that expression of Orilnc1 is controlled by RAS/RAF/MEK/ERK axis through AP1 transcription factor. Over-expression of this lncRNA has been shown in BRAF-mutant cancer cells, including melanoma cell lines. Orilnc1 silencing has sufficiently prohibited proliferation and growth of cancer cells in vitro and in vivo. Furthermore, Orilnc1 silencing could reduce cyclin E1 levels leading to induction of cell cycle arrest at G1/S phase. Thus, Orilnc1 has been identified as non-protein regulator of RAS/RAF activity and a possible target for treatment of RAS/RAF-associated malignancies [14].

KIMAT1 has been identified as a KRAS-responsive lncRNA whose expression is correlated with expression level of KRAS in lung cancer cell lines as well as clinical samples. KIMAT1 has been found to be originated from Transposable Elements and is known to be induced by MYC. This lncRNA can interact with DHX9 and NPM1 and has a crucial role in enhancing stability of these proteins. Functionally, KIMAT1 is a known target for MYC that induces lung cancer through enhancement of the maturation of oncogenic miRNAs via increasing stability of DHX9 and NPM1. Moreover, this lncRNA can preclude synthesis of tumor suppressor miRNAs through MYC-related suppression of p21. KIMAT1 silencing could suppress expression of KRAS and inactivate KRAS downstream signaling. In fact, KIMAT1 and proteins which interact with this lncRNA regulate KRAS signaling. In vivo studies have confirmed the impact of KIMAT1 silencing in blocking growth of lung cancer. Cumulatively, KIMAT1 has a role in conserving a positive feedback circuit that maintains KRAS signaling in the course of lung carcinogenesis. Moreover, interference with KIMAT1 has been suggested as a strategy to impede KRAS-associated carcinogenesis [15].

LncRNA SLCO4A1-AS1 has been found as an up-regulated lncRNA in colorectal cancer tissues through in silico assessment of two sets of microarrays data of this cancer type. Further analyses have shown correlation between up-regulation of SLCO4A1-AS1 and poor prognosis of patients with colorectal cancer. Mechanistically, SLCO4A1-AS1 promotes proliferation, migration, and invasiveness of these neoplastic cells through regulation of EGFR/MAPK pathway. SLCO4A1-AS1 silencing has significantly reduced expression levels of EGFR, KRAS, BRAF and MAP3K1 through inhibition of phosphorylation [16].

LINC01420 is another KRAS-related lncRNA which is overexpressed in pancreatic cancer tissues and cell lines. LINC01420 silencing has reduced proliferation, epithelial-mesenchymal transition (EMT) and in vivo growth of pancreatic cancer. Notably, KRAS has been identified as the mediator of pro-proliferative effects of LINC01420 in pancreatic cancer. Moreover, expression of KRAS has been shown to be regulated by MYC. LINC01420 could enhance MYC binding with KRAS promoter in the nucleus of pancreatic cancer cells. Interestingly, LINC01420 has also increased MYC levels in the cytoplasm through sequestering miR-494-3p. Cumulatively, LINC01420 facilitates progression of pancreatic cancer via releasing MYC from inhibitory effects of miR-494-3p in cytoplasm and enhancing nuclear levels of MYC-activated KRAS [17].

Table 1 shows the interaction between lncRNAs and KRAS in the context of cancer.

**Table 1:** Interaction between lncRNAs and KRAS in the context of cancer (ANTs: adjacent normal tissues)

lncRNA	Cancer subtype	Pattern of expression	Samples	Cell line	Targets/regulators	Signaling pathways	Function	References
Orilnc1	Different cancer cell lines	Up	Female nude mice	MDA-MB-231, MDA-MB-435, MCF10A, IMR90, SK-MEL-2 and LOX-IMVI	AP1, Cyclin E1	RAS-RAF-MEK-ERK signaling cascade	Orilnc1 expression enhanced cancer cell growth represses G1/S arrest	[14]
KIMAT1	Lung cancer	Up	75 tumors and matched ANTs and PDX mouse model	H1299, H460, A549, H1975, CALU1 and CALU6, lung squamous cell carcinoma cell line H520, lung fibroblasts HEL299, lung bronchial epithelial cell line HBEC3-KT	DHX9 and NPM1	KRAS signaling	KIMAT1 enhanced cancer cell survival, growth and invasion	[15]
SLCO4A1-AS1	Colorectal cancer	Up	45 pairs of CRC tissues and ANTs	HT29 and SW480	–	KRAS/EGFR/MAPK signaling pathway	SLCO4A1-AS1 induced CRC cell proliferation, migration and invasion	[16]
LINC01420	Pancreatic cancer	Up	BALB/c nude mice	HPDE6-C7, PANC-1, SW1990, HPAC, CFAPC-1, and BxPC-3 and HEK-293T	MYC/miR-494-3p	K-RAS signaling	LINC01420 enhanced cancer cell proliferation and PC EMT and induced PC tumor growth in vivo	[17]
KRAS1P	Prostate cancer	Up	–	DU145	KRAS	–	KRAS1P 3′UTR functions as endogenous microRNA decoy and plays putative proto-oncogenic role	[18]
YWHAE	Colorectal cancer	Up	6 colorectal normal and 10 colorectal cancer tissues	HT29, HCT116, SW480 and HEK293-T cells	miR-323a-3p and miR-532-5p	K-Ras /Erk1/2 and PI3K/Akt signaling pathways	YWHAE enhances cell cycle progression cell migration	[19]
PART1	Non-small cell lung cancer	Up	–	BEAS-2B, NCI-H2444, NCI-H647, A549, and NCI-H2	KRAS	–	Suppression of PART1 sensitizes wild type but not KRAS mutant cells to erlotinib	[20]
MALAT1	Prostate cancer	Up	20 pairs of PC tumor tissues and ANTs	PC3, LNCap, and DU145	miR-1	miR-1/KRAS	MALAT1 induced proliferation and inhibited apoptosis in PC cells	[21]
PCAT-1	Lung chemoresistance	Up	Tumor samples and ANTs from 96 lung cancer patients	A549, H1975	p27/CDK6	miR-182/miR-217 signaling/K-RAS	Exosomal PCAT-1 induced tumor growth and guides lymph node metastasis	[22]
lncRNA-NUTF2P3-001	Pancreatic cancer	Up	30 pancreatic cancer, 10 chronic pancreatitis and 30 noncancerous pancreatic tissues	PANC-1 and BXPC-3	miR-3923	miR-3923/KRAS pathway	lncRNA-NUTF2P3-001 enhanced viability, proliferation and invasion	[23]
TP53TG1	Pancreatic ductal adenocarcinoma (PDAC)	Up	95 pairs of PDAC tissues and ANTs	PANC-1, MIA PaCa-2, BxPC-3	miR-96 and KRAS	–	TP53TG1 promoted proliferation, inhibited apoptosis, and increased migration and invasion in PDAC cells	[24]

### Interaction between miRNAs and KRAS

miRNA are a group of non-coding RNAs that have about 22–24 nucleotides. These transcripts are single-stranded molecules that can inhibit protein synthesis through two different mechanisms. Mature miRNAs are produced via a two-step process through which primary miRNA is cleaved and loaded into the RNA-induced silencing complex. Base-pairing of miRNAs with target mRNAs can negatively regulate expression of target transcripts. Based on the degree of complementarity between miRNA and mRNA, the target mRNA is cleaved and degraded or its translation is inhibited [25].

The interaction between miRNAs and KRAS has been appraised in the context of cancer as well as non-malignant conditions. In the context of cancer, several known tumor suppressor and oncogenic miRNAs have been found to interact with KRAS. For instance, miR-217 has been demonstrated to reduce expression of KRAS in pancreatic cancer cells. This miRNA has been downregulated in the majority of pancreatic ductal adenocarcinoma tissues and in all examined cell lines of this type of cancer compared with the equivalent controls. Up-regulation of miR-217 in these cells could inhibit tumor growth and suppress anchorage-independent colony forming ability of these cells. Up-regulation of miR-217 has also decreased expression levels of KRAS protein and reduced the constitutive phosphorylation of AKT [26]. miR-96 is another tumor suppressor miRNA which directly targets the KRAS in pancreatic cancer cells. Forced over-expression of miR-96 has effectively suppressed KRAS, diminished activity of Akt signaling, and induced cell apoptosis. In vitro and in vivo experiments have verified that the tumor suppressor role of miR-96 depends on its inhibitory effects on KRAS [27]. EVI1 as a universal oncoprotein in pancreatic cancer has been shown to up-regulate KRAS levels via suppression of miR-96 [28]. Consistent with these findings, resveratrol has been shown to prevent colorectal carcinogenesis in an animal model of Kras activated cancer possibly through up-regulation of miR-96 [29]. Another experiment in colorectal cancer has shown a panel of miRNAs that precisely discriminate KRAS-mutated colorectal cancer tissues from other samples [30].

Several studies have shown the functional link between miR-143 levels and KRAS in different settings. This tumor suppressive miRNA has been shown to target KRAS in colorectal [31] and pancreatic cancer cells [32]. Down-regulation of this miRNA has been associated with poor prognosis of patients with colorectal cancer and lower progression free survival of patients receiving EGFR-targeting therapy. Yet, it has not been related with objective response to EGFR-targeting therapies [33]. A novel synthetic miR-143 has been shown to interfere with KRAS signaling network and enhance effectiveness of EGFR inhibitors [34]. Finally, miR-143 has been shown to decrease proliferation and migratory aptitude of prostate cancer cells while enhancing the cytotoxic effects of docetaxel via inhibiting KRAS [35]. Table 2 shows the interaction between miRNAs and KRAS in the context of cancer.

**Table 2:** Interaction between miRNAs and KRAS in the context of cancer (ANTs: adjacent normal tissues)

miRNA	Cancer subtype	Pattern of expression	Samples	Cell line	Targets/regulators	Signaling pathways	Function	References
miR-217	Pancreatic ductal adenocarcinoma	Down	21 pairs of PDAC specimens and ANTs samples	PANC-1, MIAPaCa-2, AsPC-1 and BxPC-3 cells	KRAS, AKT	RAS signaling pathway	MiR-217 inhibited tumor cell growth, anchorage-independent colony formation and in vivo xenograft tumor growth	[26]
miR-96	Pancreatic cancer	Down	10 pairs of pancreatic cancer tissues and ANTs Six-week-old male nude mice	MIA PaCa-2, PANC-1, and BxPC-3 and the cervical adenocarcinoma cell line HeLa	KRAS	KRAS/Akt signaling pathway	miR-96 in pancreatic cancer cells suppressed cell proliferation, migration, and invasion	[27]
miR-96	Pancreatic carcinogenesis	Down	156 PDACs, 64 IPMNs and 5 MCNs	HPDE, BxPC-3, PANC-1, PK-1, PK-8, PK-9, PK-45H, PK-45P, KLM-1 and BxPC-3	EVI1	KRAS/p27Kip1 pathway	miR-96 potently suppresses KRAS and serves as tumor suppressor in pancreatic cancer	[28]
miR-96	Colon cancer	–	APCCKO/Krasmut mice	HCT116 and SW480	KRAS,	–	Resveratrol has been shown to prevent colorectal carcinogenesis in an animal model of Kras activated cancer possibly through up-regulation of miR-96	[29]
miR‑193b	Esophageal squamous cell carcinoma	Down	53 pairs of esophageal squamous cell carcinoma tissues and ANTs	KYSE450 and TE1, and normal epithelial cell line, Het-1A	KRAS	–	miR-193b inhibited the cell growth, cell proliferation, migration and invasion, and increased the level of apoptotic cells	[36]
miR-873	Pancreatic ductal adenocarcinoma	Down	45 pancreatic tumor tissues and 45 normal tissues	MCF10A; MDA-MB-436, MDA-MB-231, MDA-MB-453, BT-20, HCC1937, SKBR3, T47D, and HEK293; PANC1, BxPC-3, MiaPaCa-2, and Capan-2	KRAS	KRAS/Akt signaling pathway	miR-873 inhibited proliferation, migration, invasion, and colony formation of PDAC cells, and induces cell apoptosis	[37]
miR-31	Colorectal cancer	Up	30 tumor specimens	Caco2, Caco2-BRAFV600E and Caco2-KRASG12V	KRAS and BRAF	–	miR-31 induced cell proliferation and migration	[38]
miR-373	Colorectal cancer	Down	30 tumor specimens	Caco2, Caco2-BRAFV600E and Caco2-KRASG12V	KRAS and BRAF	–	miR-373 inhibited cell proliferation and migration	[38]
miR-30c and miR-21	Non-small-cell lung cancer	Up	44 normal lung samples, 150 lung adenocarcinoma KRAS WT samples and 5 lung adenocarcinoma KRAS G12D samples and KRASLSL-G12D mice	H1299, A549, Calu-6, H1703, H292	NF1 and RASA1/ELK1	KRAS and NF-κB signaling	miR-30c and miR-21 promoted drug resistance and induced cell migration/invasion	[39]
miR-30c	Colorectal cancer	Down	CRC samples from 14 patients	HCT116, DLD1, SW48, HT29 and RKO CRC cells, and HEK-293	KRAS, ME1/P65		miR-30a inhibited tumor growth, migration and invasion	[40]
miR-27b-3p, miR-191-5p, miR-let7d-5p, miR-15b-5p, miR-98-5p, miR-10a-5p, and miR-149-5p	Colorectal cancer	Down	26 tumoral and 30 ANTs	–	KRAS	–	The presence of a different set of miRNAs in KRAS mutated CRC tissues could suggest their putative role as responsive molecular targets	[30]
let-7	Non-small cell lung cancer	–	74 NSCLC cases	–	KRAS	–	let-7 miRNAs is in lung cancer susceptibility	[41]
miR‐127‐3p and miR‐92a	Colorectal carcinoma	Up	Primary tumor of 60 patients with metastatic CRC	–	RSG3 and TOB1	–	Deregulated miRNAs played roles in nicotinamide adenine dinucleotide phosphate (NADPH) regeneration and G protein‐coupled receptor signaling pathways	[42]
miR-18a*	Squamous carcinoma, colon carcinoma	Down	–	Squamous carcinoma A431 cells, colon carcinoma HT-29 cells and fetal hepatic WRL-68 cells	KRAS	–	miR-18a* decreased proliferation and inhibited anchorage-independent growth of cells	[43]
miR-31-3p	Colorectal cancer	Down	Primary tumors from 149 KRAS WT patients	–	KRAS	–	miR-31-3p is a prognostic marker in patients treated with chemotherapy plus cetuximab	[44]
miR-31	Pancreatic and colorectal cancer	Up	–	HPNE cells, HCT116	KRAS, RASA1	MAPK pathway	miR-31 induced invasion and migration in PDAC lines through activation of Rho	[45]
miR-143	Colorectal cancer	Down	13 pairs of matched CRC and ANTs	Lovo cells	KRAS	ERK pathway	miR-143 functions as a tumor suppressor	[31]
miR-143-3p	Pancreatic ductal adenocarcinoma	Down	37 pairs of PDAC tissues and ANTs	MIA PaCa-2, PANC-1 and HPDE	KRAS	ERK pathway	miR-143-3p inhibited cell proliferative, migratory and invasive capacities in PDAC cells	[32]
miR-143	Colorectal cancer	Down	77 pairs of matched CRC and ANT samples	–	KRAS	–	miR-143 expression levels serve as an independent prognostic biomarker for CRC in KRAS wild-type patients	[33]
miR‐143	Colon cancer	Down	BALB/cSlc‐nu/nu (nude) mice	DLD‐1, SW48, HT29 and SW480	K‐Ras, Sos1	K‐Ras/ EGFR	miR‐143 is a tumor suppressive that inhibited proliferation and growth	[34]
miR-143	Prostate cancer	Down	Nine prostate cancer tissues	DU145 and PC3	KRAS and Cyclin D1	EGFR/RAS/MAPK pathway	miR-143 inhibits cell proliferation, migration, and improvement of chemosensitivity to docetaxel	[35]
miR-155	Colon carcinomas	Down	Cbx7+/+, Cbx7+/−, Cbx7−/− and transgenic (TG) Cbx7 mice	–	CBX7/KRAS	–	miR‐155 is a tumor suppressive gene	[46]
miR-193a-3p	Lung cancer	Down	8 pairs of NSCLC tissues and ANTs	A549 and H1975	KRAS	KRAS pathway	miR-193a-3p functions as a tumor suppressor that inhibited proliferation, viability and migration	[47]
miR-200c and miR-221/222	Colorectal cancer	Up	–	HCT116 cells and HKe3 cells	KRAS/PTEN	–	Oncogenic KRAS regulates 3D-specific molecules via miR-200c and miR-221/222	[48]
miR-29b	Colon cancer	Down	40 pairs of tumor tissues and ANTs	HT29, DLD1 and SW480	KRAS	NF-κB signaling	miR-29b-1-5p significantly suppressed cell proliferation	[49]
miR-126	Colorectal cancer	Down	–	HCT116 KRAS-WT and HCT116 KRAS-Mutant	KRAS	–	miR-126 as a selective inhibitor of the viability of KRAS-mutant cells	[50]
miR-126-3p and miR-126-5p	Colorectal cancer	Down	63 pairs of tumor tissues and ANTs	–	KRAS	–	Role of miR-126-3p and miR-126-5p related to regulation of angiogenesis, in patients with CRC treated with bevacizumab	[51]
miR-126	Colorectal cancer	Down	Colorectal tissues from 245 patients (42 noncancer:40 adenoma; 163 primary adenocarcinomas	SW480 and SW48	KRAS	–	miR-126 reduced cell proliferation, increased apoptosis and decreased accumulation of cells in the G0–G1 phase of the colon cancer cells	[52]
miR-193a-3p	Colorectal cancer	Down	70 pairs of tumor tissues and ANTs	SW480 and SW48	KRAS	EMT process	miR-193a-3p reduces the proliferation, migration and colony formation	[53]
miR-181a, miR-200c and miR-210	Colorectal cancer	Up	–	DLD-1 and DKO-4	KRAS	–	These miRNAs are possibly associated with CRC development	[54]
miR-134	Majority of glioblastoma	Down	–	U87, U373, A172, T98G, SNB-19, and SF-767	KRAS and STAT5B	KRAS/STAT5B	miR-134 inhibits cancer cell and stem-cell proliferation and survival	[55]
miR-134	Renal cell carcinoma	Down	24 pairs of tumor specimens and ANTs	786-O, caki-1, 769-P, HEK-293T and ACHN	KRAS	KRAS-related MAPK/ERK	miR-134 could also inhibit migration and invasion by blocking EMT	[56]
miR-134	Glioma	Down	63 glioma tissue samples	U251	KRAS	ERK pathway	miR-134 inhibited glioma cell proliferation and invasion	[57]
miR‑98	Retinoblastoma	Down	RB samples from 60 patients	WERI-Rb-1, Y79 and SO-RB50	IGF1R	IGF1R/k‑Ras/Raf/MEK/ERK signaling pathway	miR‑98 suppress cell growth, migration and invasion	[58]
Let-7a	Colorectal carcinomas	Down	Tissue from 172 patients	–	KRAS	–	Higher let-7a levels were significantly associated with better survival outcomes	[59]
miR-193b	Pancreatic ductal adenocarcinoma	Down	Pancreatic tissue samples from 10 patients	MIA PaCa-2, PANC-1, AsPC-1 and BxPC-3, and hTERT-HPNE	KRAS	AKT and ERK pathways	miR-193b inhibits pancreatic cancer cell growth and proliferation	[60]
miR-206	Pancreatic ductal adenocarcinoma	Down	SCID mice	PANC-1, PANC10.05, BxPC-3, MiaPaca-2, CFPAC-1, Colo357 and Capan-1	KRAS and ANXA2	NF-κB signaling	miR-206 inhibits cell cycle progression, cell proliferation, migration and invasion	[61]
miR-21	Non-small-cell lung cancer	Up	Transgenic mice	–	KRAS	Ras/MEK/ERK pathway	MiR-21 drives tumorigenesis through inhibition of negative regulators of the Ras/MEK/ERK pathway and inhibition of apoptosis	[62]
miR-3923	Pancreatic cancer	Down	Pancreatic tissue samples from 30 pancreatic cancers, 10 chronic pancreatitis and 30 noncancerous pancreatic tissues	PANC-1 and BXPC-3		miR-3923/KRAS pathway	miR-3923 inhibits viability, proliferation and invasion	[23]
miR-489	Pancreatic ductal adenocarcinoma	Down	NOD/SCID mice	BxPC-3 and PANC-1	ADAM9 and MMP7	KRAS-NF-κB-YY1	miR-489 inhibits the migration and metastasis	[63]
miR-155	Pancreatic cancer	Up	–	Capan2, Aspc1, Panc1 and BxPC3	KRAS and Foxo3a	MAPK and NF-κB pathway	miR-155 promotes pancreatic cancer cell proliferation	[64]
miR‑337	Colorectal cancer	Down	54 paired CRC tissues and ANTs	LoVo, HCT116, HT29, SW480, SW620	KRAS	AKT and ERK signalling pathways	miR-337 inhibits cell proliferation, invasion and increases apoptosis	[65]
miR-217	Osteosarcoma	Up	–	Human osteosarcoma 143B cell line	KRAS	miR-217-KRAS axis	miR-217 downregulation led to the loss of enhanced cisplatin sensitivity	[66]
miR-193a-3p	Lung cancer	Down	10 female athymic nude mice	MDA-MB-231, HeyA8 and SKOV3.ip1 cells	KRAS	–	miR-193a-3p functions as a tumor suppressor by inhibiting K-Ras	[67]
miR-768-3p	Brain tumor and lung cancer	Down	19 brain tissue from patients	H520, A549, H661, H441, astrocyte cell line	KRAS	–	miRNA-768-3p inhibits K-ras and suppresses metastasis	[68]

The interaction between miRNAs and RAS pathway has also been appraised in the context of cardiac hypertrophy. Sayed et al. have reported that a group of miRNAs are differentially and temporally altered in the course of cardiac hypertrophy. Notably, the muscle-specific miRNA miR-1 has been shown to be decreased in very early phase of this process, continuing through day 7 following aortic constriction-associated hypertrophy of heart. This miRNA could inhibit expressions of RasGAP, Cdk9, fibronectin, and Rheb [69].

### Interaction between circRNAs and KRAS

CircRNAs are a group of non-coding RNAs with an enclosed circular conformation that is shaped by either typical spliceosome-mediated or lariat-type splicing [70]. This circular configuration protects circRNAs from effects of RNases, thus circRNAs have more stability than linear RNAs [71]. Circ_GLG1 is a KRAS-related circRNA which is considerably over-expressed in colorectal tissues compared with nearby normal tissues. Silencing of circ_GLG1 in colorectal adenoma carcinoma cells could inhibit viability of tumor cells. Moreover, circ_GLG1 silencing reduces proliferation, invasiveness, and migratory potential of these cells. These processes could be reversed by transfection of miR-622 antagonist. Circ_GLG1 could promote KRAS expression through serving as a miR-622 sponge. Cumulatively, circ_GLG1/miR-622/KRAS axis has been found to participate in the pathogenesis of colorectal cancer [72].

CircITGA7 is another KRAS-related circRNA whose expression is considerably decreased in CRC tissues and cells in association with cancer progression. Forced over-expression of circITGA7 could suppress growth and metastatic potential of colorectal cancer cells. On the other hand, circITGA7 silencing could promote malignant behavior of these cells both in vitro and in vivo. Functionally, circITGA7 acts as a negative modulator of the Ras signaling pathway through binding with to miR-370-3p to antagonize its inhibitory effects on neurofibromin 1. Moreover, circITGA7 increases expression of ITGA7 via inhibiting RREB1 through the Ras pathway [73].

Another study has shown global down-regulation of circRNAs in DLD-1 and DKO-1 colorectal cancer cells (containing KRAS mutant allele) compared to DKs-8 cells (containing only wild type alleles of KRAS), representing an extensive influence of mutant KRAS on expression profile of circRNAs. Additional experiments in KRAS mutant HCT116 cells and KRAS wild type HKe3 cells have confirmed this observation. Notably, circRNAs have been detected in cancer-derived extracellular-vesicles in higher abundance than cells. This finding implies their potential as tumor biomarkers [74]. Table 3 shows the interaction between circRNAs and K-RAS in the context of cancer.

**Table 3:** Interaction between circRNAs and K-RAS in the context of cancer (ANTs: adjacent normal tissues)

circRNAs	Diseases	Pattern of expression	Samples	Cell lines	Targets/regulators	Signaling pathways	Function	References
FAT1; HIPK3; ARHGAP; MAN1A2; RHOBTB3; RTN4; SMARCA5	Colon cancer	Down	–	DLD-1, DKO-1 cells, DKs-8 cells	KRAS	–	circRNAs may serve as promising cancer biomarkers	[74]
circITGA7	Colorectal cancer	Down	69 pairs of colorectal cancer samples and ANTs	s (SW480, RKO, Caco-2, SW620, LoVo, HCT116 and DLD1	ITGA7	Ras pathway	circITGA7 represses the proliferation and metastasis of CRC cells via inhibiting the Ras signaling pathway and inducing the transcription of ITGA7	[73]
Circ_GLG1	Colorectal cancer	Up	40 pairs of CRC tissues and ANTs	HCT116, SW620, and DLD1 cells	miR-622	Ras pathway	circ_GLG1 promoted tumor cell viability, proliferation, invasion, and migration	[72]
circFNTA	Bladder cancer	Up	41 cancer tissues and matched ANTs	SVHUC, BCa cell lines T24, J82, 5637, and UMUC3	miR-370-3p	Ras pathway	circFNTA induced cell invasion and cisplatin chemo-resistance	[75]
Circ-MEMO1	Non-small cell lung cancer	Up	52 pairs of SCLC tissue samples and ANTs	H1650, PC9, H1299, and A549	miR-101-3p	miR-101-3p/KRAS Axis	Circ-MEMO1 induced the progression and aerobic glycolysis of lung cancer cells	[76]

The interaction between circRNAs and KRAS has also been assessed in hyperglycemic conditions. A circRNA from human umbilical vein endothelial cell exosomes has been shown to affect senescence process in the vascular smooth muscle cells in hyperglycemic niche. CircRNA-0077930 has been found to serve as a sponge for miR-622 to increase expression of KRAS. Exosome-mediated transfer of circRNA-0077930 could induce senescence of smooth muscle cells through the above-mentioned mechanism. Besides, this circRNA could increase LDH activity and reduce superoxide dismutase activity in these cells [77].

## Discussion

The data reviewed in the current manuscript show the close interaction between KRAS oncoprotein and several non-coding RNAs, particularly in the context of lung [15], pancreatic [17] and colorectal cancers [16]. In fact, these three types of cancer are the main types of malignancies in which KRAS has been found to be epigenetically modulated by non-coding RNAs. Glioma, retinoblastoma, osteosarcoma, bladder cancer, prostate cancer and esophageal cancer are other types of cancers in which the interaction between KRAS and non-coding RNAs has been verified.

The interaction between KRAS and non-coding RNAs not only affects cell proliferation and apoptosis [16], but also mediates EMT [17] and stemness [55]. LINC01420 [17], miR-134 [55] and miR-193a-3p [53] are examples of KRAS-interacting non-coding RNAs that partake in this process. KRAS-interacting transcripts also affect response of cancer cells to chemotherapeutics such as docetaxel [35] and cisplatin [75]. Most notably, a number of these transcripts have been found to determine prognosis and course of malignancy among affected individuals.

The data summarized in this review shows the combinatorial effect as well as balancing effects of different non-coding RNAs on KRAS regulation in cancers. In fact, KRAS is regulated by multiple non-coding RNAs, and many of the non-coding RNAs are relevant at a time in cancers. No study has revealed any organ or environment specificity in expression of these non-coding RNAs. Instead, most of above-mentioned non-coding RNAs have similar roles in the pathogenesis of several different cancers, indicating their universal effects in regulation of KRAS independent from tissue type.

LncRNAs that regulate expression of KRAS mostly exert this function through serving as sponges for miRNAs. MALAT1/miR-1, PCAT-1/miR-182/miR-217 and lncRNA-NUTF2P3-001/miR-3923 are examples of miRNA/lncRNAs that regulate expression of KRAS. Similarly, circRNAs can serve as molecular sponges for KRAS-associated miRNAs. Circ_GLG1/miR-622, circFNTA/miR-370-3p and circ-MEMO1/miR-101-3p axes have been shown to regulate expression of KRAS in colorectal, bladder and lung cancer cells. Therefore, a complex functional network between different classes of non-coding RNAs is involved in the regulation of KRAS levels in cancers. Identification of other elements of this multifaceted network can provide novel insight about the carcinogenesis and facilitate design of more appropriate targeted therapies.

Besides, it is worth mentioning that non-coding RNAs can act either upstream or downstream of KRAS. For instance, lncRNA Orilnc 1, circRNA FAT1 and HIPK3 are downstream targets of KRAS, but not the regulators of KRAS. Several other non-coding RNAs have been shown to regulate expression of KRAS.

Several mechanisms participate in KRAS regulation by lncRNAs. For instance, lncRNAs act as sponges for miRNAs that target KRAS. Moreover, lncRNAs have functional associations with numerous regulatory apparatuses, including chromatin remodeling elements, transcription factors, splicing apparatus and nuclear trafficking regulators [78]. Through these interactions, they can also regulate expression of KRAS. Modulation of establishment of G4 elements in the promoter region of KRAS is another possible mechanism by which lncRNAs can influence expression of KRAS. For instance, KRASIM, the microprotein coded by the lncRNA NCBP2-AS2 has been found to suppress expression of KRAS and inhibit ERK signaling in hepatocellular carcinoma cells [79].

The impact of KRAS-related non-coding RNAs on cellular activities has also been assessed in the context of hyperglycemia and cardiac hypertrophy. However, data regarding their impact on other non-malignant conditions is scarce.

## Conclusion

Future studies are needed to find whether the presence of mutations in KRAS can affect the interaction between non-coding RNAs and this oncoprotein. Moreover, the impact of these non-coding RNAs on resistance to targeted therapies should be more clarified. Finally, the relative contribution of KRAS mutations and dysregulation of KRAS-related non-coding RNAs in the pathogenesis of human cancer should be clarified. This field will benefit from the development of new techniques, such as single cell sequencing and CRISPR-CAS9 gene editing.

## Data Availability

Data sharing not applicable to this article as no datasets were generated or analysed during the current study.
